# Detection of disease-associated α-synuclein in the cerebrospinal fluid: a feasibility study 

**DOI:** 10.5414/NP300796

**Published:** 2014-08-18

**Authors:** Ursula Unterberger, Ingolf Lachmann, Till Voigtländer, Walter Pirker, Anna S. Berghoff, Katharina Flach, Uta Wagner, Aline Geneste, Armand Perret-Liaudet, Gabor G. Kovacs

**Affiliations:** 1 Institute of Neurology, Medical University of Vienna, Vienna, Austria,; 2 AJ Roboscreen GmbH, Leipzig, Germany,; 3 Department of Neurology,; 4 resent address: Department of Medicine I, Clinical Division of Oncology, Medical University of Vienna, Vienna, Austria, and; 5 Neurobiologie, CMRR, Hospices Civils de Lyon, Université Lyon, Lyon, France

**Keywords:** α-synuclein, bead-assay, cerebrospinal fluid, Parkinson’s disease dementia, dementia with Lewy bodies, synucleinopathy

## Abstract

With the aim to evaluate the significance and reliability of detecting disease-specific α-synuclein in the cerebrospinal fluid (CSF) we developed an ELISA and bead-assay. We used a commercial antibody (5G4) that does not bind to the physiological monomeric form of α-synuclein, but is highly specific for the disease-associated forms, including high molecular weight fraction of β-sheet rich oligomers. We applied both tests in CSF from a series of neuropathologically confirmed α-synucleinopathy cases, including Parkinson’s disease dementia (PDD) and dementia with Lewy bodies (DLB) (n = 7), as well as Alzheimer’s disease (n = 6), and control patients without neurodegenerative pathologies (n = 9). Disease-specific α-synuclein was detectable in the CSF in a subset of patients with α-synuclein pathology in the brain. When combined with the analysis of total α-synuclein, the bead-assay for disease-specific α-synuclein was highly specific for PDD/DLB. Detection of disease-associated α-synuclein combined with the total levels of α-synuclein is a promising tool for the in-vivo diagnosis of α-synucleinopathies, including PDD and LBD.

## Introduction 

Neurodegenerative disorders are characterized by the formation and deposition of abnormal isoforms of physiologically occurring proteins primarily in the brain of affected individuals [1]. In the case of the Parkinson’s disease (PD) and dementia with Lewy-bodies (DLB) [2], this is α-synuclein, a presynaptic protein, which under pathological conditions is deposited in neurons and neuronal processes in the form of Lewy bodies and Lewy neurites [3]. α-Synuclein is an extremely useful marker for the neuropathological diagnosis and staging of the aforementioned disorders. It is therefore most desirable to make use of it not only in autopsies, but also for early clinical diagnosis, while the patient could still potentially benefit from the result. 

Especially the in-vivo detection of disease-associated α-synuclein (d-α-syn) has important clinical implications. In particular, due to the frequent co-occurrence of Lewy body pathology with Alzheimer’s disease (AD) pathology, it is crucial to differentiate it from pure AD forms. This has significant therapeutic relevance, since patients with the former are sensitive to treatment with neuroleptics. Therefore, the analysis of d-α-syn could improve biomarker-diagnosis of dementing illnesses. In particular, detection of d-α-syn in body fluids may precede clinical symptoms or abnormalities in classical neuroimaging. To date, the analysis of total α-synuclein (t-α-syn) in cerebrospinal fluid (CSF) has been described in several publications [4, 5, 6, 7, 8, 9, 10, 11, 12] (see [13] for a review), while only single studies have dealt with the detection of disease-associated forms, including oligomers, in human plasma or CSF [7, 14]. However, there is no commercially available kit for the detection of d-α-syn in body fluids. 

We recently described an antibody against α-synuclein (named 5G4), which shows high specificity for the disease-associated forms, including high molecular weight fraction of β-sheet rich oligomers, while no binding to primarily disordered oligomers or monomers was observed [15, 16]. Furthermore, we have demonstrated that d-α-syn deposits in the ependymal layer of the ventricles and aqueduct [15] in PD and DLB, thus it is likely that it can be detected in the CSF. In the present study, we developed an ELISA and bead-assay for the detection of d-α-syn in CSF using this antibody. Our major aim was to evaluate whether d-α-syn is detectable in the CSF or not. Therefore, we investigated the CSF in a cohort of neuropathologically examined cases, including 7 with Lewy body-related pathology. 

## Materials and methods 

### Case selection and CSF samples 

The present study included 22 cases of neuropathologically confirmed cases, among them patients with α-synucleinopathy with Lewy-related pathology (7 cases; 2 of them clinically classified as Parkinson’s disease dementia, PDD [17] and 5 as DLB), with AD (6 cases), and controls without neurodegenerative pathologies (9 cases) (Table 1). The CSF samples were collected during the surveillance for human prion disease, which includes follow-up of patients with progressive dementia. Neuropathological evaluation excluded prion disease in the cases included in the present study and confirmed the diagnoses mentioned above. CSF was evaluated for protein 14-3-3 and the remaining samples were analyzed whether d-α-syn can be detected in the CSF or not. This allowed us the investigation of CSF in combination with autopsy material from the same patient. Due to the fact that CSF examination is not a routine procedure during the diagnosis of PD, this strategy enabled us to provide a basis for longitudinal in-vivo studies. 

For this study, systematic neuropathological examination [18] was carried out including immunohistochemistry for d-α-syn to confirm or rule out the presence of Lewy body pathology. Indeed, we excluded α-synuclein pathology in all cases with AD and controls without neurodegenerative pathologies (Table 1). All CSF samples were collected and transported to the lab at ambient temperature and centrifuged at 4,100 × g for one minute at 10 °C immediately upon arrival. Supernatants were stored in 100 µL aliquots in polypropylene tubes at –80 °C. The study was approved by the local ethics committee (Nr. 397/2011). 

### ELISA analysis of t- and d-α-syn in the CSF 

ELISA highsorb plates white (Greiner, Frickenhausen, Germany) coated with antibodies 5G4 or 10D2 [16] were used for incubation with 100 µL of CSF diluted 1 : 1 in phosphate buffered saline (PBS) pH 7.7 containing 0.05% Tween 20, 3% bovine serum albumin (BSA), 5 mM EDTA and 10 mM PefaBlock (dilution buffer). Controls and synthetic standards containing target antigens were incubated in parallel to patient samples for 24 hours at 2 – 10 °C. Plates were washed 5 times, and detection antibodies (horseradish-peroxidase (HRP)-conjugated anti-α-synuclein 10D2 for 5G4-coated plates, or rabbit anti-α-synuclein (AJ Roboscreen GmbH, Leipzig, Germany) for 10D2-coated plates, respectively, both at 0.5 µg/mL in dilution buffer), were added for 90 minutes at room temperature. All plates were washed 10 times, and 10D2-coated plates were additionally incubated with an HRP-conjugated anti-rabbit IgG antibody (Dianova, Hamburg, Germany). Antibody reactions were developed using ECL substrate FEMTO (Pierce, Rockford, IL, USA), and luminescence counts were measured using an Ascent reader (Thermo, Rockford, IL,USA). 

### Bead-assay for d-α-syn in the CSF 

For 5G4 coating, 1 mg streptavidin-coated magnetic beads (DynaBead M280-Streptavidin, LifeTechnologies, USA) were incubated with 20 µg of biotinylated antibody 5G4 in dilution buffer (same as for the ELISA) for 30 minutes at room temperature. Beads were collected with magnetic power for 3 minutes, supernatants discarded, and beads washed three times in dilution buffer. Coated beads could be stored at 4 °C for up to 4 weeks. 

For immunoprecipitation and detection, Innupure C16 machine with an adapted software (AJ) was used. 250 µL of CSF diluted 1 : 1 in dilution buffer, as well as synthetic standards were transferred onto 0.2 mg 5G4-coated beads and incubated for 24 hours at 4 °C. During the whole incubation time, samples were mixed continuously. After 3 washes in dilution buffer, HRP-conjugated 10D2 was applied for 2 hours at room temperature. After a final washing step, beads were transferred in enhancer solution (ECL substrate FEMTO) onto white ELISA plates, peroxide was added, and luminescence measured by the Ascent reader. 

### Statistical analysis 

For all quantitative parameters (age, t-α-syn, d-α-syn/ELISA, d-α-syn/bead assay, as well as the ratio of t-α-syn to d-α-syn/ELISA or d-α-syn/bead-assay), Kruskal-Wallis and Mann-Whitney (M-W) tests were used to compare the diagnostic groups of patients. Spearman correlation test was performed to correlate the time lapse between lumbar puncture and death. Differences were considered as statistically significant at p < 0.05. For statistical analyses, we used SPSS Statistics (V21.0, SPSS Inc., Chicago, IL, USA). 

## Results 

**Figure 1:**
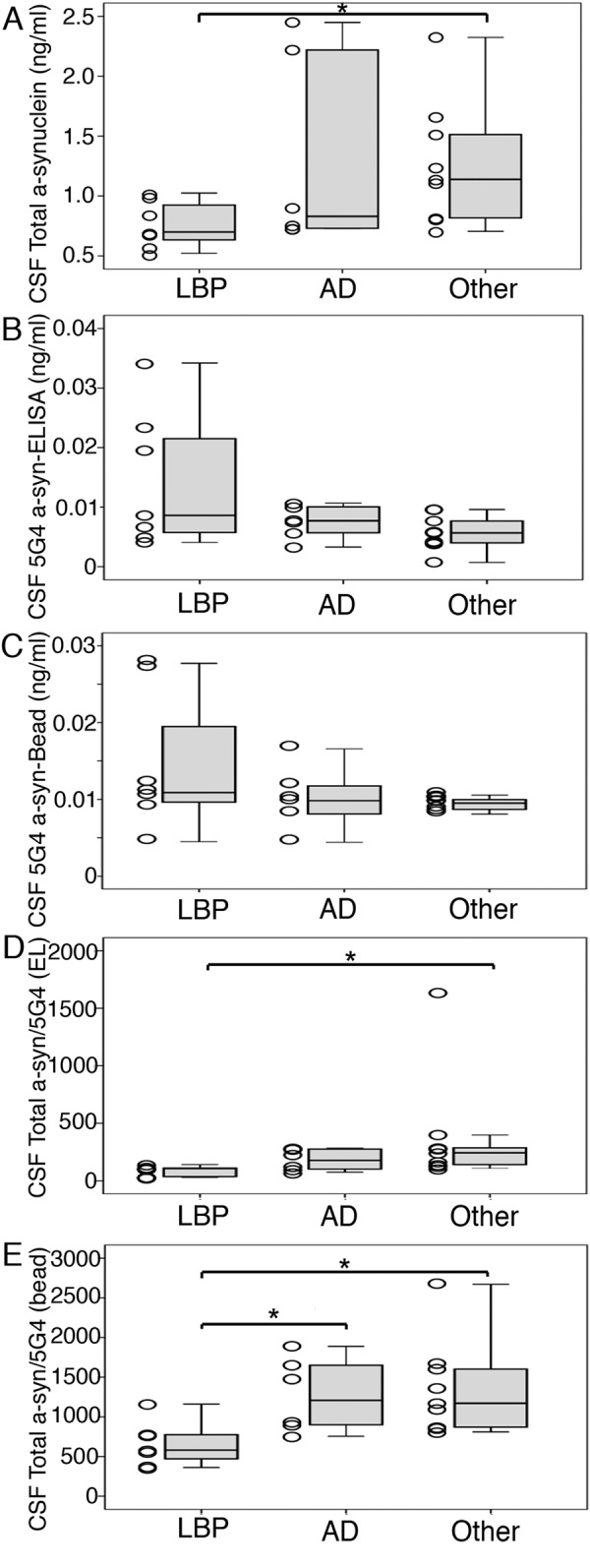
Box-plot and scatter plot demonstration of the levels of total (A) and disease-specific α-synuclein by ELISA (B) and bead-assay (C) in the CSF in cases with Lewy-body pathology (LBP), Alzheimer`s disease (AD), and in controls without neurodegenerative pathologies. Results for the ratio of disease-specific to total α-synuclein in each sample are shown in D and E.

In addition to t-α-syn, using the ELISA kit and the bead-assay, we were indeed able to detect high levels of d-α-synuclein in the CSF in a subset of patients with α-synucleinopathy (PDD and DLB). The levels of t-α-syn tended to be lower in CSF samples from PDD/DLB patients as compared to AD and were significantly lower than in controls without neurodegenerative pathologies (M-W test p = 0.016) (Figure 1A). By contrast, in both the ELISA kit and the bead-assay, d-α-syn was – on average – higher in PDD/DLB than in the other groups (Figure 1B, C). There was a lack of distinct neuropathological features of PDD/DLB cases with high values. In particular, we found no correlation with Braak stage of Lewy body pathology. Neither CSF protein levels and storage time, nor contamination with erythrocytes (as evaluated in accordance to a protocol developed by partners of BIOMARKAPD consortium in the frame of EU Joint Programme – Neurodegenerative Disease Research), which was present in one CSF sample of each group of patients, had any influence on the results. 

The ratio of d-α-syn to t-α-syn (t-α-syn/d-α-syn) was calculated for each sample. For the ELISA results, the ratio was significantly different only when comparing PDD/DLB with controls (M-W test: p = 0.002) (Figure 1D). However, using the bead-assay, the ratio was significantly lower in the PDD/DLB group in comparison to both AD (M-W test: p = 0.022) and controls without neurodegenerative pathologies (M-W test: p = 0.002) (Figure 1E). Using the bead-assay based ratio t-α-syn/d-α-syn, only 1 false positive (1/15) and 1 false negative result was found (1/7). We did not find an effect of the time lapse between the lumbar puncture and the time of death either in the pooled cohort of all cases, or in separate groups (PDD/DLB, AD, and controls). 

## Discussion 

In this feasibility study, we addressed the question whether d-α-syn can be detected in the CSF in patients with proven Lewy body pathology-related dementias. Previous studies have analyzed oligomeric forms of α-synuclein in plasma and CSF [7, 14]; however, we present here the first study in a cohort of eventually neuropathologically confirmed patients. We used an antibody against d-α-syn (5G4) in an ELISA-kit, and a recently developed immunoprecipitation assay (bead-assay), respectively. We found that the bead-assay provided much more reliable results with generally lower background, which was probably due to combination of higher sample volume and intensive washing used for the bead-assay. 

Most importantly, we observed a significant difference in the mean values of the ratio of d-α-syn to t-α-syn between α-synucleinopathy (PDD/DLB) patients and controls without neurodegenerative pathologies, and between α-synucleinopathy (PDD/DLB) and AD patients. Although the levels of d-α-syn detected by ELISA or bead-assay alone were not highly specific for PDD/DLB, it is remarkable that in our small series of seven α-synucleinopathy patients, four showed a value of the ratio of d-α-syn to t-α-syn below the lowest value detected in AD patients and controls without neurodegenerative pathologies (Table 1). We cannot explain the difference of these levels among the PDD/DLB patients by a specific neuropathological pattern, since immunomorphology of α-synuclein and Braak stages were not different in these cases. 

As reported previously by others [4], we observed a decrease of t-α-syn in the CSF of patients with PDD/DLB. However, this alone was not highly specific for the differentiation of this group from AD or other, non-neurodegenerative disorders. Extremely high levels of d-α-syn were detectable in three PDD/DLB patients with the ELISA, and in two PDD/DLB patients with the bead-assay. Neuropathology did not differ from other subjects with α-synucleinopathy. Overall, the usefulness of the ratio of d-α-syn to t-α-syn is most likely to be explained by the decrease in t-α-syn, while d-α-syn is elevated or remains the same, which leads to a more obvious difference of the ratio of these two values in α-synucleinopathy (PDD/DLB) patients as compared to controls. 

It is extremely interesting to compare our findings with recent findings reported in the field of AD. Recently, it was suggested that the ratio of oligomeric aβ to aβ42 in CSF reached a higher level of statistical significance between AD patients from healthy normal controls compared to the standard aβ42 assay alone or to the aβ oligomers assay alone [19]. It must be highlighted that in these two disorders, it was shown that oligomers (Aβ and α-synuclein) were found to be the cytotoxic entities. Moreover, it is highly suspected that part of the decrease of total CSF levels in α-synuclein of LBD, and aβ42 peptide of AD patients could be explained by deposition of these proteins in plaques and Lewy bodies [19]. Our observations, using a neuropathologically confirmed cohort of PDD/DLB patients, support the results of a recent study performed on clinically classified PD subjects [20]; there the ratio of oligomers/total α-synuclein was calculated and found to be increased (in our study the ratio was calculated the opposite way). 

In summary, we show that d-α-syn can indeed be detected in the CSF of patients clinically classified as PDD/DLB and neuropathologically showing Lewy-related α-synuclein pathology using an antibody highly specific for d-α-syn. We are aware that further investigations (“validation study”) on larger cohorts will be needed to evaluate the exact specificity and sensitivity of this test. However, the present feasibility study supports the concept that combined detection of d-α-syn and t-α-syn might be a promising tool for the in-vivo differentiation of patients with PDD/DLB from other types of dementia. Since d-α-syn is present in the brains of individuals with PD or multiple system atrophy, the present study supports the notion [20] for examining the CSF in these patients for diagnostic purposes, including the evaluation of progression or the risk for the development of dementia. 

## Acknowledgments 

The authors acknowledge the excellent technical assistance of I. Heinrich, F. Rashidian, E. Drobna, M. Kainz, and S. Zimmermann. This study was partly supported by the European Commission’s 7th Framework Programme under GA No 278486, “DEVELAGE” (for GGK). 

## Conflict of interest 

Ingolf Lachmann, Katharina Flach, and Uta Wagner are employees of the diagnostic company AJ Roboscreen. Antibody 5G4 is patented by AJ Roboscreen, inventors are Gabor G. Kovacs, Ingolf Lachmann, Awad A. Osman, and Uta Wagner. UU, TV, WP, ASB,AG, APL report no conflict of interest. 

**Table 1:** Cases included in the study.

Case	Sex	Age	Disease	Neuropathology
1*	m	78	DLB	LBP: Braak stage 4. BBVI; CAA. Argyrophilic grain disease.
2*	w	80	PDD	LBP: Braak stage 4. BBV; CAA. Limbic TDP-43 proteinopathy.
3	w	80	DLB	LBP: Braak stage 4. Progressive supranuclear palsy (tauopathy). Vascular encephalopathy. Limbic TDP-43 proteinopathy.
4*	m	73	DLB	LBP: Braak stage 5. BBII. Metabolic gliosis. Meningeosis leukemica.
5	m	72	DLB	LBP: Braak stage 6. BBV. Vascular encephalopathy.
6	w	81	DLB	LBP: Braak stage 6. BBV; CAA.
7*	w	73	PDD	LBP: Braak stage 6. BBIV; CAA.
8	w	85	AD	BBVI. Thalamic microinfarction.
9	w	82	AD	BBVI. CAA.
10	w	86	AD	BBVI. CAA. Bilateral infarction occipital lobe. Cerebellar infarction.
11	w	82	AD	BBVI. Severe atherosclerosis with infarcts in basal ganglia, occipital lobe and cerebellum.
12	w	84	AD	BBIV. Infarction right parietal lobe.
13	w	81	AD	BBVI. CAA.
14	m	48	C	Chronic meningoencephalitis (neurolues). Metabolic gliosis.
15	w	49	C	Binswanger encephalopathy.
16	w	78	C	Angiotropic B-cell Lymphoma.
17**	m	71	C	Viral polioencephalitis (TBE).
18	w	83	C	Multi-infarct encephalopathy.
19	m	59	C	Viral polioencephalitis (TBE).
20	w	68	C	Viral polioencephalitis. Acute ischemic/hypoxic encephalopathy.
21	m	40	C	Multi-infarct encephalopathy. Metabolic gliosis.
22	m	56	C	Limbic encephalitis.

DLB = dementia with Lewy bodies; PDD = Parkinson’s disease dementia; LBP = Lewy body pathology; BB = Braak & Braak stageing of neurofibrillary degeneration (I – VI); AD = definite Alzheimer’s disease; CAA = cerebral amyloid angiopathy; TBE = tick borne encephalitis; C = control. *These patients had lower value of the combined total/5G4 bead assay ratio than the lowest of the controls indicated by **.
